# Disease activity, autoantibodies, and inflammatory molecules in serum and cerebrospinal fluid of patients with Systemic Lupus Erythematosus and Cognitive Dysfunction

**DOI:** 10.1371/journal.pone.0196487

**Published:** 2018-05-03

**Authors:** Alí Duarte-García, Juanita Romero-Díaz, Sandra Juárez, Alba Cicero-Casarrubias, Hilda Fragoso-Loyo, Carlos Núñez-Alvarez, Luis Llorente, Jorge Sánchez-Guerrero

**Affiliations:** 1 Department of Medicine, Division of Rheumatology,Mayo Clinic. Rochester, Minnesota, United States of America; 2 Robert D. and Patricia E. Kern Center for the Science of Health Care Delivery, Mayo Clinic. Rochester, Minnesota United States of America; 3 Department of Immunology and Rheumatology, Instituto Nacional de Ciencias Médicas y Nutrición Salvador Zubirán, México City, México; 4 Department of Neurology, Instituto Nacional de Ciencias Médicas y Nutrición Salvador Zubirán, México City, México; 5 Department of Medicine, Division of Rheumatology. Mount Sinai Hospital and University Health Network. Toronto, Ontario, Canada; University of Patras School of Medicine, GREECE

## Abstract

**Objective:**

To determine if cognitive dysfunction in patients with systemic lupus erythematosus (SLE) derives from an inflammatory process with continuing disease activity, and increased levels of autoantibodies and inflammatory molecules in serum and cerebrospinal fluid (CSF).

**Methods:**

100 randomly selected patients participating in an inception SLE cohort were studied. At entry into the cohort, a standardized medical history and extensive laboratory tests profile, including autoantibodies were completed. Follow-up occurred every 3–6 months with assessment of lupus characteristics, comorbidities, and treatment. After a mean follow-up of six-years, cross-sectional evaluation of cognitive function was done with standardized tests, and in a subset of patients an extended profile of autoantibodies, cytokines and chemokines was measured in serum and CSF.

**Results:**

At enrollment into the cohort, patients were 26.4±8.2 years of age and lupus duration 5.3±3.7 months. Moderate/severe cognitive dysfunction was diagnosed in 16 patients; in comparison to patients with normal cognitive function, they had lower education 9 vs. 12 years (P = 0.006), higher body mass index 26.7 vs. 24.3 (P = 0.03), positive IgG anticardiolipin antibodies 50% vs 18% (P = 0.009), and a higher median number of concomitant NPSLE syndromes 3 vs. 1, (P = 0.04). The prevalence of cardiovascular-risk factors, other auto-antibodies, lupus activity, treatment, and incidence of critical events did not differ. In serum and CSF, the levels of autoantibodies, cytokines and chemokine were similar, only CCL2 was elevated in CSF [886.1 (374.9–1439.7) vs. 515.8 (3.2–1958.2) pg/mL, P = 0.04].

**Conclusion:**

Scant evidence of inflammation in SLE patients with cognitive dysfunction was observed. Only a higher prevalence of IgG anticardiolipin antibodies in serum and increased levels of CCL2 in CSF were detected.

## Introduction

Cognitive dysfunction is one of the 19 neuropsychiatric syndromes affecting patients with systemic lupus erythematosus [1]. The reported prevalence 15–81%, severity, course, and impact of cognitive dysfunction are greater in patients with SLE than in the general population and patients with rheumatoid arthritis, suggesting that SLE itself contributes to cognitive dysfunction [2–8]. In comparison to rheumatoid arthritis, severe cognitive dysfunction develops in SLE more often, despite demographic features, cardiovascular risk factors, mean daily steroid dosage, prevalence and mean scores of anxiety and depression are similar, suggesting that generic risk factors for impairment play a more relevant role in SLE [2,6–8].

Cognitive dysfunction is not restricted to SLE or systemic autoimmune diseases, it also develops after acute and chronic health disorders e.g., major surgery, coronary-artery bypass surgery, critical illnesses, systemic hypertension, diabetes mellitus, hemodialysis among others [9–14]. Other variables associated to cognitive impairment in the general population include age and cerebrovascular disease [9–10].

In lupus, cognitive dysfunction has been associated to antiphospholipid antibodies, anti-NR2 antibodies, lower education, disease activity, chronic damage, hypertension, stroke, and corticosteroid use, however these results are inconsistent [2,5,6].

Despite cognitive dysfunction is one of the most frequent neuropsychiatric syndromes in SLE, affecting social functioning, work capacity, and employment [7,15,16], its pathogenesis remains elusive and likely multifactorial. In addition, there are no specific recommendations to prevent its onset and no defined treatment once it is diagnosed.

In this study, we aimed to define if cognitive dysfunction results from persistent inflammation characterized by ongoing disease activity, the presence of high levels of autoantibodies, cytokines, and chemokines in serum and CSF, or is an epiphenomenon resulting from chronic damage, concomitant critical medical events, and/or diverse comorbidities observed in SLE patients.

## Materials and methods

### Study participants

One hundred ambulatory patients, participating in a prospective cohort of SLE of recent-onset at enrollment, were studied. The study was approved by the Institutional Review Board “Institutional Committee of Biomedical Research” (IRE– 508), and all subjects provided written informed consent to participate.

#### Lupus cohort

In October 1999, an inception cohort of patients aged ≥16 years, who were within 12 months of accrual ≥4 ACR classification criteria for systemic lupus erythematosus [7–18] was assembled as part of an international consortium for the study of atherosclerosis and neuropsychiatric manifestations [19–22].

At entry, patients had a standardized medical history, physical examination, and laboratory tests, including routine chemical analyses, total cholesterol, high-density lipoprotein cholesterol (HDL), low-density lipoprotein cholesterol (LDL), triglycerides, Lp(a) lipoprotein by cholesterol content, apo B, homocystein, high-sensitivity C-reactive protein (hs-CRP), serum complement (C_3_ and C_4_), and autoantibodies: antinuclear, anti-double-stranded DNA, anti-Sm, anti-RNP/Sm, anti-RNP, anti-SSA/SSB, anticardiolipin, and anti-β2-glycoprotein. The positivity of antibodies was defined per the laboratory cut-off values.

Every 3–6 months, patients are seen in the lupus clinic for medical care, and assessed for disease activity using the SLE disease activity index 2000 update (SLEDAI-2K) [23], medications use and dose, following a standardized protocol. Every year, the information is updated, including chronic damage accrual using the Systemic Lupus International Collaborating Clinics damage index (SDI) [24], co-morbidities, traditional cardiovascular risk factors, and a blood sample is drawn. Since cardiovascular and neuropsychiatric events are primary outcomes of the cohort, they are continuously monitored.

Clinical information is stored in a database containing demographic, anthropometric, lifestyle habits, medical family history, obstetric variables, and lupus information. Two rheumatologists perform all assessments.

#### Cognitive dysfunction study

After a mean of six-year of follow-up, 100 out of 210 patients participating in the cohort were selected using a computer-generated randomization list and invited to participate in this cross-sectional study.

All participants provided additional medical information using an ad-hoc questionnaire, had a physical examination, and laboratory tests. Cognitive function was assessed using the following tests: Trail Making test, Digit Span, California Verbal Learning test, Rey-Osterrieth complex figure test, the Stroop Color-Word test, WAIS III letter-number sequencing, Animal naming test, Controlled Oral Word association, WAIS-R/III digit symbol substitution test, Grooved pegboard test, and WAIS-R/III similarities. Tests were grouped in seven cognitive domains: memory, attention/executive function, visuospatial, motor, psycho-motor speed, language, and problem solving. These tests are consistent with the neuropsychological test battery recommended by the ACR [1]. We did not include any Depression Index. A certified neuropsychologist, blinded to clinical information but knowing the patient’s demographic characteristics applied and graded the cognitive tests. Cognitive Dysfunction was defined as scores ≥2 SD below the mean of age-matched normative data on at least 2 cognitive domains.

#### Autoantibodies, cytokines and chemokines in serum and cerebrospinal fluid

Within one month of the cognitive function testing, patients were invited to participate in the analysis of autoantibodies and inflammatory molecules in serum and CSF. In this phase of the study, participants had a blood sample drawn, and a lumbar puncture guided with ultrasound to obtain a sample of CSF. Serum and CSF samples were immediately frozen at -70°C until processing. The analyses included the following autoantibodies [anti-NMDA, nucleosomes, ribosomal-P, RNP-70, ds-DNA, β_2_-glycoprotein-I IgG and IgM, anticardiolipin IgG and IgM], cytokines and chemokines [Interferon-alpha (IFN-α, Interleukin-6 (IL-6) and CCL2, CXCL10, CXCL8, and CCL-19].

Anti-dsDNA, anti-cardiolipin, and anti-β2 glycoprotein-I antibodies were detected by immunoenzymatic assay (EIA) as per the manufacturer’s recommendations (The Binding Site, Birmingham, UK). Similarly, anti-ribosomal P, nucleosome, and anti-RNP-70 antibodies were detected by EIA (Orgentec Diagnostika, Germany). In all instances serum samples were diluted 1:100 and CSF samples were tested undiluted. Anti-NMDAR antibodies were detected by ELISA as previously described [25]. Briefly, DWEYS peptide was adsorbed to microtiter plates in 0.1M NaHCO3 (pH 8.6) overnight at 4°C. Serum was assayed at 1:50 and 1:100 dilution and developed with an enzyme tagged antibody to human IgG. Antinuclear antibodies of the IgG isotype were detected by indirect immunofluorescence as per the manufacturer’s recommendations (The Binding Site). Serum samples were diluted 1:40 and CSF samples were tested undiluted.

Levels of IFN-α, IL-6, CCL2, CXCL8, CXCL10 and CCL19 were measured using Luminex bead-based technology as per the manufacturer’s recommendation (Invitrogen, Carlsbad, CA, USA) in all patients. The detection limit of the experimental system was 3.2 pg/ml for all cytokines and chemokines.

#### Statistical analyses

Risk factors were summarized in terms of median (minimum–maximum) or mean ± standard deviation, and absolute frequencies (%). Cumulative lupus activity during the entire period of follow-up was summarized as SLEDAI-2K areas under the curve (AUC) and time-adjusted means [26]. Moderate/severe activity was defined as cumulative months with SLEDAI-2K score ≥7. Univariate comparisons of patients with and without cognitive dysfunction were conducted by means of the Student-t test or Mann-Whitney U test, and Chi-square or Fisher exact test, as appropriate. Logistic regression models including variable with a P value ≤0.10 in the univariate analysis, were run to account for potential confounders. P value was set at ≤0.05, two-tailed. The SPSS (SPSS Inc, Chicago, IL, version 11.5) statistical package was used.

## Results

The distribution of demographic, anthropometric, and lupus characteristics, comorbidities, and cardiovascular risk-factors at enrollment into the cohort did not differ between participating and non-participating patients, except that participating patients had lower levels of homocysteine, hsCRP, lower prevalence of anti-SSA antibodies (P ≤ 0.01), and higher frequency of the immunologic criteria (P = 0.03) (data not shown).

At enrollment into the cohort, participating patients had mean age 26.4 ± 8.2 years, and lupus duration 5.3 ± 3.7 months. At assessment of cognitive function, mean age of patients was 32.6 ± 7.9 years, disease duration 6.2 ± 3.5 years, and mean level of education 12.4 ± 3.4 years.

### Cognitive dysfunction

Cognitive dysfunction was diagnosed in 16 patients, age 24–57 and disease duration 1.2–11.0 years. Four patients were diagnosed within the first five year of SLE diagnosis and 12 patients after it. In comparison to patients with normal cognitive function, they had lower education 9 vs. 12 years (P = 0.006), higher body mass index 26.7 vs. 24.3 (P = 0.03), less frequently arthritis 63% vs 89% (P = 0.001), and higher prevalence of IgG anticardiolipin antibodies 50% vs 18% (P = 0.009) (Tables 1 and 2); tended to be older, median age 35 vs. 31 years (P = 0.09), post-menopausal 21% vs. 5%, (P = 0.07), with higher median SLICC-DI score, excluding neuropsychiatric items, 1 vs 0 (P = 0.08), and more frequent renal involvement 81% vs 55% (P = 0.06). The prevalence of traditional cardiovascular-risk factors including levels of lipids, glucose, homocysteine, hs-CRP, and the Framingham Risk-Score, was similar between both subgroups (Table 1).

**Table 1:** Demographic characteristics and cardiovascular risk-factors of SLE patients at screening of cognitive dysfunction*.

Variable	Cognitive Dysfunction N = 16	No Cognitive Dysfunction N = 84	P Value
Age — *yr*	35 (24–57)	31 (18–51)	0.09
Females — *n (%)*	14 (88)	79 (94)	0.31
Education, yrs	9 (3–16)	12 (6–19)	0.006
Postmenopausal— *n (%)*	3 (21)	4 (5)	0.07
Body mass index, kg/m^2^	26.7 (20.3–38.1)	24.3 (16.7–40.5)	0.03
Waist (cm)	86 (71–101)	84 (62–114)	0.36
Waist/hip ratio	0.85 (0.78–0.92)	0.84 (0.59–1.33)	0.75
Currently smoking — n (%)	0	12 (14)	0.21
Hypertension^†^ — n (%)	6 (38)	19 (23)	0.22
Diabetes — n (%)	0	2 (2)	1.00
Cholesterol, mg/dl			
Total	183 (127–218)	179 (105–381)	0.98
Low-density lipoprotein	100 (59–139)	98 (55–255)	0.98
High-density lipoprotein	45 (31–89)	47 (20–97)	0.91
Triglycerides, mg/dL	142.5 (74–358)	116 (32–431)	0.15
Lp(a) lipoprotein, mg/dL	16.5 (2–196)	10.6 (2–140)	0.23
Apo B, mg/dL	82.15 (54.7–135)	79.4 (38.8–192)	0.64
Glucose, mg/dL	83 (55–92)	81 (49–112)	0.51
Homocystein, mg/dL	11.1 (3.5–17.9)	9.45 (5.5–22.4)	0.14
Ultrasensitive C reactive protein, mg/dL	2.3 (0.22–9.9)	2.0 (0.04–9.9)	0.55
Framingham risk score, baseline	1 (1–3)	1 (1–4)	0.60

*Data are expressed as mean (SD), number (percentage), or median (minimum–maximum).

^†^ Hypertension defined as systolic blood pressure ≥140 mmHg or diastolic blood pressure ≥90 mmHg.

No difference in disease activity was observed at enrollment into the cohort, throughout the course of lupus, and at assessment of cognitive function; neither the length of moderate/severe disease activity was different; however, patients with cognitive dysfunction tended to accrue more chronic damage. The prevalence of anti-dsDNA, anti-Sm, anti-RNP/Sm, anti-SSA, anti-SSB, anti-ribosomal P, and anti-β_2_-glycoprotein-I antibodies, as well as lupus anticoagulant in serum did not differ (Table 2).

**Table 2:** Disease characteristics, clinical manifestations, and autoantibodies of SLE patients as per the presence of cognitive dysfunction (CD)*.

Variable	Cognitive Dysfunction N = 16	No Cognitive Dysfunction N = 84	P Value
***SLE characteristics***			
Age at diagnosis, yrs	26.5 (17–51)	24 (14–46)	0.09
Disease duration, yrs	8.2 (1.2–11.0)	5.7 (0.5–11.1)	0.24
Length of follow-up, yrs	7.7 (1.2–10.0)	5.2 (0.5–10.2)	0.39
Number of SLE criteria	6 (4–9)	6 (4–9)	0.81
SLEDAI-2K score at baseline†	7 (0–14)	6 (0–27)	0.83
SLEDAI-2K at screening of CD	4 (0–14)	4 (0–24)	0.86
SLEDAI-2K-AUC, mean	16.0 (5.6–49.7)	14.6 (0.7–39.2)	0.18
SLEDAI-2K adjusted mean	4.9 (0.5–11.0)	4.1 (0.2–16.7)	0.58
Length of moderate/severe disease activity (SLEDAI-2K score ≥ 7), months	6 (0–17)	3 (0–80)	0.66
SLICC‡ Damage Index score at screening	1 (0–4)	0 (0–4)	0.08
Anti-dsDNA antibodies IU/mL	12.6 (4.7–164)	15.4 (0–1338)	0.56
C3 Levels mg/dL	96.6 (37.6–143)	77.1 (10.5–184)	0.57
C4 Levels mg/dL	11.2 (1.7–29.7)	12.4 (1.7–47.7)	0.91
***SLE manifestations***			
Malar rash — n (%)	8 (50)	34 (41)	0.58
Discoid rash, — n (%)	1 (6)	9 (11)	1.00
Photosensitivity, — n (%)	6 (38)	25 (30)	0.56
Oral ulcers, — n (%)	6 (38)	33 (39)	1.00
Arthritis, — n (%)	10 (63)	75 (89)	0.01
Serositis, — n (%)	8 (50)	34 (41)	0.58
Renal disorder, — n (%)	13 (81)	46 (55)	0.06
Neurological disorder, — n (%)	2 (13)	4 (5)	0.25
Hematological disorder, — n (%)	11 (69)	66 (79)	0.52
***Autoantibodies at enrollment***			
Anti-dsDNA antibodies, — n (%)	6 (38)	37 (44)	0.79
Anti-Sm antibodies, — n (%)	9 (56)	44 (52)	0.79
Anti-RNP/Sm antibodies, — n (%)	5 (31)	35 (42)	0.58
Anti-SSA antibodies, — n (%)	8 (50)	39 (46)	1.00
Anti-SSB antibodies, — n (%)	5 (31)	23 (27)	0.77
Anti-phospholipid antibodies, — n (%)			
IgM anti-cardiolipin antibodies	6 (38)	20 (24)	0.35
IgG anti-cardiolipin antibodies	8 (50)	15 (18)	0.009
Any anti-cardiolipin antibodies	9 (56)	28 (33)	0.09
IgM anti-β2GPI antibodies	4 (25)	16 (19)	0.73
IgG anti-β2GPI antibodies	4 (25)	13 (15)	0.46
Any anti-β2GPI antibodies	6 (38)	24 (29)	0.55
Lupus anticoagulant	2 (20)	10 (12)	0.41

*Data are expressed as mean (SD), number (percentage), or median (minimum–maximum).

† Systemic lupus erythematosus disease activity index 2000

‡ Systemic lupus erythematosus international collaborating clinics-damage index. Scores exclude neuropsychiatric items.

The incidence of critical events during the evolution of lupus was similar for all-hospitalizations, emergency and intensive care unit hospitalizations, length of hospitalization, use of vasopressors and antibiotics between patients with and without cognitive dysfunction. In addition, the use and cumulative dose of steroids, immunosuppressants, anti-malarials, and low-dose aspirin did not differ. (Table 3).

**Table 3:** Hospitalizations and treatment of SLE patients according to the presence of cognitive dysfunction*.

Variable	Cognitive Dysfunction N = 16	No Cognitive Dysfunction N = 84	P Value
***Hospitalization data***			
Hospitalization — ever— n (%)	10 (63)	34 (41)	0.16
Length of hospitalization in days, median (range)	30 (7–141)	17.5 (5–91)	0.31
Number of hospitalizations, median (range)	2 (1–4)	2 (1–5)	0.34
Due to medical indication — n (%)	9 (90)	27 (79)	0.65
Due to surgical indication — n (%)	1 (10)	4 (12)	1.00
Both — n (%)	0 (0)	3 (9)	1.00
Elective hospitalization— n (%)	1 (10)	2 (6)	0.54
Emergency hospitalization — n (%)	9 (56)	32 (38)	0.54
Critical care hospitalization— n (%)	2 (20)	4 (12)	0.60
Vasopressor use — ever — n (%)	2 (20)	5 (15)	0.65
Antibiotics use — ever — n (%)	7 (70)	24 (71)	1.00
***Treatment — n (%)***			
Prednisone use, ever	16 (100)	83 (99)	1.00
Cumulative dose of Prednisone, gr	25.9 (10.9–61.1)	17.7 (1.1–64.9)	0.19
Azathioprine use, ever	15 (94)	65 (77)	0.18
Cyclophosphamide use, ever	9 (56)	29 (35)	0.16
Any immunosuppressant	15 (94)	67 (80)	0.29
Antimalarials, ever	8 (50)	58 (69)	0.16
Low-dose aspirin, ever	9 (56)	45 (54)	1.00

*Data are expressed as mean (SD), number (percentage), or median (minimum–maximum).

Eight (50%) patients with cognitive dysfunction had at least another NPSLE syndrome (seizures 4, stroke 3, lupus headache 3, polyneuropathy 2, cranial neuropathy 1, movement disorder 1), in comparison to 31 (27%) without cognitive dysfunction (P = 0.40); however, the median number of NPSLE syndromes in patients with cognitive dysfunction was higher 3 (1–5) vs. 1 (1–3), (P = 0.04).

In the logistic regression analysis, adjusting for age and gender, IgG aCL antibodies was the single independent risk factor identified, OR 4.93 (95% CI 1.36–17.89, P < 0.02), while education showed a protective effect, OR 0.78 (95% CI 0.64–0.95, P < 0.02).

### Analyses of autoantibodies, cytokines and chemokines

Matched samples of serum and CSF were available from 10 patients with and 30 patients without cognitive dysfunction. In serum, the levels of all antibodies, including anti-NMDA antibodies, were similar between groups. Also, the levels of IL-6, IFNα, CCL2, CXCL8, CXCL10, and CCL19, did not differ (Table 4).

**Table 4:** Levels of autoantibodies, cytokines and chemokines in serum of patients with and without cognitive dysfunction*.

Antibodies, cytokines, chemokines	Cognitive Dysfunction N = 10	No Cognitive Dysfunction N = 30	P Value
Anti-NMDA U/mL	6.3 (4–100)	6.3 (3–100)	0.24
Nucleosome U/mL	106 (7.2–674.6)	87.2 (7.1–700)	0.97
Ribosomal P U/mL	8.05 (4.7–17.2)	6.4 (4.9–286.2)	0.46
RNP70 U/mL	7.7 (5.9–302.2)	6.7 (5.6–212.9)	0.56
Anti-dsDNA IU/mL	17.1 (10–209.2)	18.5 (10.4–109.9)	0.45
β2GP1-IgG U/mL	5.3 (1.8–16.7)	2.9 (1.7–259)	0.3
β2GP1-IgM U/mL	3.6 (2.2–64.6)	3 (2.1–58.9)	0.69
aCL-IgG UGPL	6.8 (4.7–42.7)	5.7 (42–246.5)	0.39
aCL-IgM UMPL	5.9 (3.4–238.2)	7.7 (3.5–123.5)	0.83
CCL2 pg/mL	567.7 (335.2–1039.7)	487.5 (258.9–2160.4)	0.51
CXCL10 pg/mL	578.7 (283.5–1000)	501.9 (184.3–2596.4)	0.87
CXCCL8 pg/mL	66.3 (13.3–848.2)	102.6 (16.7–1590.3)	0.40
IL-6 pg/mL	31.2 (3.2–214.9)	61.9 (3.2–1559.3)	0.32
INF alpha pg/mL	57.6 (3.2–473.2)	34.9 (3.2–1867.7)	0.66
CCL19 pg/mL	132.5 (92.6–1380.7)	113.9 (41.6–468.7)	0.13

*Data are expressed as median (minimum–maximum).

Anti-NMDA = Anti-*N*-methyl-D-aspartate receptor, Anti-RNP70 = Anti-ribonucleoprotein 70, IL-6 = interleukin 6.

In CSF, the levels of anti-NMDA, anti-ribosomal-P, anti-dsDNA, anti-Sm, anti-RNP70, anti-nucleosomes, IgG/IgM anticardiolipin, and IgG/IgM anti-β_2_GPI were comparable. Also, the levels of IL-6, IFNα, CXCL8, CXCL10, and CCL19 were similar; only CCL2 was elevated in patients with cognitive dysfunction [886.1 (374.9–1439.7) vs. 515.8 (3.2–1958.2) pg/mL, P = 0.04] (Table 5).

**Table 5:** Levels of autoantibodies, cytokines and chemokines in cerebrospinal fluid of patients per the presence of cognitive dysfunction*.

Antibodies, cytokines, chemokines	Cognitive Dysfunction N = 10	No Cognitive Dysfunction N = 30	P Value
Anti-NMDA U/mL	3 (1.5–5)	3 (1.5–12.5)	0.26
Nucleosome U/mL	12.4 (5.2–294.7)	11.5 (5.8–332.8)	0.56
Ribosomal P U/mL	4.8 (4.7–5.7)	5 (4.7–300.5)	0.08
RNP70 U/mL	3.8 (3.6–7.8)	4 (3.6–6.3)	0.52
Anti-dsDNA IU/mL	9.9 (8.9–11.4)	10.7 (8.6–54.4)	0.08
β2GP1-IgG U/mL	4.5 (2.4–9.0)	4.9 (2.1–14.5)	0.57
β2GP1-IgM U/mL	1.9 (1.8–14.5)	1.9 (1.8–2.3)	0.87
aCL-IgG UGPL	2.9 (2.7–5.0)	3.0 (2.7–37.2)	0.35
aCL-IgM UMPL	2.3 (2.0–150.0)	2.2 (1.9–3.5)	0.44
CCL2 pg/mL	886.1 (374.9–1439.7)	515.8 (3.2–1958.2)	0.04
CXCL10 pg/mL	813.3 (260.9–4330.9)	711.05 (3.2–2841.6)	0.39
CXCL8 pg/mL	21.7 (9.8–39.2)	19.3 (3.2–52.6)	0.55
IL-6 pg/mL	3.2 (3.2–11.8)	3.2 (3.2–4.3)	0.74
INF alpha pg/mL	11.4 (3.2–38.7)	8.8 (3.2–29.7)	0.14
CCL19 pg/mL	43.4 (9.8–115.2)	34.8 (9.7–169.6)	0.41

*Data are expressed as median (minimum–maximum).

Anti-NMDA = Anti-*N*-methyl-D-aspartate receptor, Anti-RNP70 = Anti-ribonucleoprotein 70, IL-6 = interleukin 6.

## Discussion

We detected cognitive dysfunction in 16 percent of young patients with SLE of short evolution Cognitive function was assessed with a battery of neurocognitive tests evaluating seven domains. We aimed to identify patients with moderate/severe cognitive dysfunction to prevent misclassification, and the prevalence observed falls within the range reported [5].

Single-factor analyses revealed that low education, higher BMI, lower prevalence of arthritis, higher number of NPSLE syndromes, IgG anti-cardiolipin antibodies in serum, and increased levels of CCL2 in cerebrospinal fluid were associated with cognitive dysfunction. However, multivariate analysis showed that only low-level education and IgG anticardiolipin antibodies in serum were associated to cognitive dysfunction.

Our results are consistent with previous studies where lower education and higher BMI have been associated with lower cognitive scores in the general population [27–29]. In SLE, IgG anticardiolipin antibodies is one of the variables most consistently associated to cognitive dysfunction [5,6,30,31]. Persistently elevated levels of IgG anticardiolipin antibodies are associated with significantly poorer performance in cognitive function and cognitive deterioration [30,31]. The association of higher BMI and anticardiolipin antibodies with cognitive dysfunction supports the notion of a vascular mechanism underlying cognitive dysfunction. Higher BMI is also an independent predictor of cerebral atrophy [29].

Our hypothesis that cognitive dysfunction results from persistent inflammation characterized by ongoing disease activity, with presence of autoantibodies, cytokines, and chemokines in serum and CSF derives from the dissimilar results of former studies [5,6,27]. A positive association between disease activity and cognitive dysfunction was reported in the Lupus Outcomes Study [5], where disease activity was assessed cross-sectional, using the Systemic Lupus Activity Questionnaire (SLAQ), a self-completed or telephone administrated survey [32]. Other studies did not find an association between lupus activity and cognitive dysfunction [6,27]. We found no difference in clinical disease activity between patients with and without cognitive dysfunction at enrollment into the cohort, throughout the course of lupus, and at assessment of cognitive function; neither the length of moderate/severe disease activity differed. The contrasting result with the Lupus Outcomes Study may derive from the live and longitudinal assessment of disease activity in our study.

Several reports described that anti-dsDNA antibodies cross-reacting with the NR2 glutamate receptor, mediate apoptotic death of neurons when they gain access to CSF, mediating non-thrombotic and non-vasculitic abnormalities of the CNS. These antibodies were detected in the CSF from a patient with SLE who had progressive cognitive decline and in vivo neuronal death, and in vitro primary neuronal death of mouse brain into which the CSF was injected [33]. However, two large studies did not observe an association between serum anti-NR2 antibodies and cognitive dysfunction [27,34]. Since the strongest association of anti-NR2 antibodies with NPSLE syndromes is when they are measured in CSF not in serum [35], we analyzed their association in serum and CSF, nevertheless the results were negative for all the antibodies in both specimens.

Previously, we reported that SLE patients with inflammatory neuropsychiatric manifestations present in CSF high levels of IL-6 and the chemokines CXCL8, CCL5, CXCl9, and CXCL10 during the acute event and the levels decrease significantly after improvement [36]. In addition, among seven patients with refractory lupus headache these inflammatory molecules reached the same levels as in patients with other major NPSLE syndromes and were significantly elevated than in patients with non-NPSLE and patients with non-autoimmune diseases [37]. In the current study, the levels in CSF of CXCL10, CXCL-8, IFN-α, and CCL-19 were similar in patients with and without cognitive dysfunction, only CCL2 levels were slightly elevated. Previous reports suggest that chemokines, particularly CCL2, may have another function in the damaged CNS that is distinct from its role in proinflammatory events [38,39]. SLE patients show neurologic damage over the long-term, which manifests itself as a decrease in brain volume on magnetic resonance imaging studies and as a worsening of cognitive function clinically [40,41]. Even though this worsening is greater in patients with a history of NPSLE, it is also found in patients without previous NP manifestations [42,43]. There is no recognized etiology of these alterations; however, it is possible that this slight, but persistent, increase in chemokine levels in the CSF of SLE patients elicits this damage by maintaining a chronic low-level response [36].

In this regard, previous reports have indicated that in patients with subtle cognitive impairment, there is an increase in CCL2 [44,45]. Even though current knowledge does not enable us to establish a cause-and-effect relationship between the increase in CCL2 levels and cognitive dysfunction the link between them is suggestive that this indeed could be the case.

Overall, the assessment of autoantibodies, cytokines and chemokines in serum and cerebrospinal fluid of patients with cognitive dysfunction, did not reveal an active inflammatory process as in other NPSLE syndromes [36,46].

As there are no validated biomarkers of lupus activity to date, we tried to ponder representative inflammatory molecules that have been associated with disease activity. Thus, we included representatives of cytokines and chemokines families, endothelial activation and soluble surface molecules. However, measurement of these potential biomarkers did not show any association.

Patients with medical and surgical critical illnesses are at high risk for long-term cognitive impairment [11,13]. Since patients with lupus frequently suffer these conditions, we analyzed the rate of hospitalizations including emergency and intensive care unit, length of hospitalization, severe infections, and use of vasopressors, but no association was observed. Unfortunately, we were unable to assess history of delirium which is a risk factor for cognitive deficit. Since this is the first study assessing critical illnesses as risk factors for cognitive dysfunction in lupus, we cannot compare our results.

Our results do not support a definitive inflammatory mechanism underlying cognitive dysfunction in patients with SLE. Yet, its onset predominantly late during the evolution of the disease, the association with older age, higher body mass index, antiphospholipid antibodies, and cumulative number of previous NPSLE syndromes suggests that it derives from chronic damage, probably of vascular origin. Several studies support this proposal: in the Lupus Outcomes Study, antiphospholipid antibodies, hypertension, and history of stroke were associated with cognitive impairment [5]. Also, a strong association between hypertension, antiphospholipid antibodies, accumulated damage, and magnetic resonance imaging with the number of mental functions and domains impaired was reported [6]. In a late middle-aged general population, hypertension was associated with cognitive decline over 6 years [9]. In our study, despite the longitudinal assessment of traditional cardiovascular risk-factors, we found no association with cognitive dysfunction. Neither, with use/dose of medications including corticosteroids, low-dose Aspirin, and anti-malarials.

Our study has potential limitations. Neuropsychological assessment as well as measurement of autoantibodies and inflammatory molecules in CSF was cross-sectional after six-years of follow-up, so we cannot dismiss that the levels of antibodies as well as inflammatory molecules may fluctuate overtime. As compared to other major NPSLE manifestations with an acute onset, cognitive dysfunction presents gradually, so if an inflammatory mechanism triggers cognitive dysfunction and disappears later, we were unable to detect it; therefore, we cannot fully exclude an inflammatory mechanism in the pathogenesis of cognitive dysfunction. However, we were able to assess prospectively the level of disease activity along the follow-up and found no difference between groups. We did not perform imaging studies, so we cannot assess changes in the neuroanatomical structure such as cerebral atrophy, infarctions, or white matter hyperintensities and their potential involvement in cognitive dysfunction. We did not assess the presence of depression, an important confounder when assessing Cognitive Function; however, some studies failed to find that depression or anxiety are primary causes of cognitive impairment in SLE [27,47–49]. The number of patients with moderate/severe cognitive dysfunction in our study is small due to the characteristics of the population studied such as age and disease duration. The small sample might limit the robustness of the results and conclusions, as well as the power to identify other factors associated; nevertheless, their relevance should be less than the weight imposed by the factors identified in the study. In another study, we studied seven patients with refractory headache and were able to define the inflammatory nature of this NPSLE manifestation [37], so we do not think that a type II error explains our negative results. This is a single center study with limited ethnic variation; one must be cautious about extrapolating these results to all lupus patients.

Strengths of our study include the stringent longitudinal assessment of independent variables including cardiovascular risk factors, cardiovascular and neuropsychiatric events, lupus characteristics, and autoantibodies. We studied participants in an inception cohort of young patients within the early years of the disease, so we were able to assess cognitive dysfunction within the early stages minimizing any confounding effect of age-related or non-lupus related variables. We assessed prospectively disease activity along the follow-up, as well as factors not previously explored such as the incidence of critical illnesses. To the best of our knowledge, this is the first study addressing specifically the levels of diverse autoantibodies, cytokines and chemokines both in serum and CSF in relation to cognitive dysfunction.

Based on the results of this study, we conclude that the evidence of an inflammatory mechanism underlying cognitive dysfunction is scants; therefore, its detection should not instigate to modify the immunosuppressive treatment.

## Supporting information

S1 Dataset Copy of database cognitive. (XLS)
